# Supplementary material for: Archival influenza virus genomes from Europe reveal genomic variability during the 1918 pandemic
Source: Nat Commun. 2022 May 10;13:2314. doi: 10.1038/s41467-022-29614-9 (PMC9090925; doi:10.1038/s41467-022-29614-9)
Supplement: Supplementary file 1 — Supplementary Information [file 41467_2022_29614_MOESM1_ESM.pdf]

Supplementary Information

Archival influenza virus genomes from Europe reveal genomic variability during the 1918 pandemic

Livia V. Patrono[1, 2]†, Bram Vrancken[3]†, Matthias Budt[4]†, Ariane Düx[1, 2], Sebastian Lequime[5], Sengül Boral[6], M. Thomas P. Gilbert[7, 8], Jan F. Gogarten[1, 2], Luisa Hoffmann[4], David Horst[6], Kevin Merkel[1, 2], David Morens[9], Baptiste Prepoint[2,10], Jasmin Schlotterbeck[2], Verena J. Schuenemann[11], Marc A. Suchard[12,13,14], Jeffery K. Taubenberger[15], Luisa Tenkhoff[4], Christian Urban[11], Navena Widulin[16], Eduard Winter[17], Michael Worobey[18], Thomas Schnalke[16], Thorsten Wolff[4], Philippe Lemey[3], Sébastien Calvignac-Spencer[1, 2]*

Affiliations:

[1]Epidemiology of Highly Pathogenic Microorganisms, Robert Koch Institute, Berlin, Germany.

[2]Viral Evolution, Robert Koch Institute, Berlin, Germany.

[3]Laboratory of Clinical and Evolutionary Virology, Department of Microbiology, Immunology and Transplantation, Rega Institute, Katholieke Universiteit Leuven, Leuven, Belgium.

[4]Unit 17 Influenza and other Respiratory Viruses, Robert Koch Institute, Berlin, Germany.

[5]Cluster of Microbial Ecology, Groningen Institute for Evolutionary Life Sciences, University of Groningen, Groningen, The Netherlands.

[6]Institute for Pathology, Charité, Berlin, Germany.

[7]Center for Evolutionary Hologenomics, The GLOBE Institute, University of Copenhagen, Copenhagen, Denmark.

[8]University Museum, NTNU, Trondheim, Norway.

[9] Office of the Director, National Institute of Allergy and Infectious Diseases, Bethesda, Maryland.

[10]Département de Biologie, Ecole Normale Supérieure, PSL Université Paris, Paris, France.

[11]Institute of Evolutionary Medicine, University of Zurich, Zurich, Switzerland.

[12]Department of Biostatistics, Fielding School of Public Health, University of California, Los Angeles, Los Angeles, CA, USA.

[13]Department of Biomathematics, David Geffen School of Medicine, University of California, Los Angeles, Los Angeles, CA, USA.

[14]Department of Human Genetics, David Geffen School of Medicine, University of California, Los Angeles, Los Angeles, CA, USA.

[15]Viral Pathogenesis and Evolution Section, Laboratory of Infectious Diseases, National Institute of Allergy and Infectious Diseases, Bethesda, Maryland.

[16]Berlin Museum of Medical History, Charité, Berlin, Germany.

[17]Pathological-anatomical collection in the Narrenturm, Natural History Museum of Vienna, Vienna, Austria.

[18]Department of Ecology and Evolutionary Biology, University of Arizona, Tucson, Arizona.

*Correspondence to: calvignacs@rki.de

†These authors contributed equally to this work

Supplementary Note 1: Histopathology

We performed histopathological analyses of the MU-162, BE-572 and BE-576 lung specimens. For conventional histopathology, slides of lung tissue were stained with hematoxylin and eosin. A gram staining was also performed to identify gram-positive bacteria. Sample MU-162 was characterized by severe purulent, partially confluent bronchopneumonia with small hemorrhages and edema without clear histomorphological evidence of bacterial colonization (**Supplementary Figs. 1a and 1b**). Sample BE-572 was characterized by severe purulent, partially hemorrhagic bronchopneumonia with alveolar edema and evidence of bacterial colonization by gram-positive cocci (**Supplementary Figs. 1c and 1d**). Sample BE-576 was characterized by severe pulmonary alveolar edema with pronounced hemorrhages, emphysema and first stages of acute bronchopneumonia with evidence of bacterial colonization by gram-positive cocci (**Supplementary Figs. 1e and 1f**).

Supplementary Note 2: Evaluation of human RNA preservation

Fully formed RNA virus particles are built to protect the integrity of the viral genome under various conditions for days or even months[1]. This might also affect RNA recovery from formalin-fixed tissue, due to a better RNA protection prior to fixation in comparison to the endogenous RNA. Hence, we assessed the endogenous human RNA preservation by mapping all reads to a full human transcriptome reference (**Supplementary Table 1**). Strikingly, most of these fragments show a significantly lower average length than the fragments mapping to the IAV reference (Extended Data Fig. 3). The smaller fragments of human endogenous RNA compared to the viral RNA are a first indication of the protective function of the virion in RNA recovery from formalin-fixed wet specimens. The length of fragments mapping only to the transcriptome varied greatly between the samples and within independent extractions from the same sample. This might be due to varying time between patient death and formalin fixation, the exact formalin composition (e.g. buffered or unbuffered formalin and formalin concentration), different penetration of the fixative in different parts of the tissue, or remaining gDNA fragments which leads to RNA fragmentation during the RNase H treatment of the rRNA depletion step.

Sample	Mapped reads	Endogenous human reads [%]	Average fragment length [nt]
MU-162	1452871	6.53	62.7
BE-576	779471	4.28	59.8
MU-162/2	263258	1.01	118.3
BE-576/2	1795933	4.60	64.6
BE572	123143	0.28	104.3

Supplementary Table 1. Mapping of the influenza-positive samples to a human transcriptome reference. Shown are the results for independently generated libraries.

Supplementary Note 3: Genomic comparison of 1918 influenza viruses

Together with the available BM and CU sequences, we used these influenza genomes from Germany to assess the genomic diversity of: (i) strains simultaneously involved in local transmission (BE-572 and BE-576), (ii) strains circulating in Europe (MU-162, BE-572 and BE-576) and North America (BM and CU), (iii) strains circulating during the pre-pandemic (BE-572 and BE-576) and pandemic peak period (BM and CU).

The two Berlin genomes sampled on June 28th 1918, for which only a portion of the genome could be compared, differed from each other at only two (non-synonymous) nucleotide positions in the HA gene (**Fig. 1b**). These positions were however polymorphic in BE-576, with the minor variant being identical to BE-572. At a country/continent scale, BE-572 and MU-162 on one hand and BM and CU on the other differed both by 22 and 15 single nucleotide polymorphisms (SNPs; 12 non-synonymous, all genes but HA, NS and MP and 7 non-synonymous, all genes but NP and PB2, respectively, **Supplementary Table 2 and Supplementary Fig. 4**). The four pairwise comparisons of European and North American strains identified 22-43 SNPs, with the two best genomes (MU-162 and BM) differing at 43 positions, 18 of which coding for amino acid changes (all genes but HA). When comparing the pre-pandemic European strain BE-572 with pandemic peak strains BM and CU, we identified 29 and 22 SNPs (11 non-synonymous, all genes but HA and NS genes, and 8 non-synonymous, all genes but NS genes, respectively).

We acknowledge that we could not fully disentangle these comparisons (the two Berlin genomes are not complete, and the two accurately dated genomes from the pre-pandemic peak period are from European specimens, while the two accurately dated genomes from the pandemic peak are from North America) and our sample size was very small.

	BM	CU	MU-162	BE-572
BM	-			
CU	15 (11)	-		
MU-162	43 (36)	33 (30)	-	
BE-572	29	22	22	-

Supplementary Table 2. Number of differences between the highest quality genomes. Numbers in () represent the differences counted in the positions covered by BE-572 (12023 nt). BE-576 was not included in these comparisons because of its lower quality. A 70 nt fragment in PA with a signal of recombination (8 SNPs, 1 coding) was excluded from this comparison.

An aspect that stood out from this comparison is that we identified a 70 nt stretch of the PA of BM comprising 8 SNPs that shows a high degree of identity to IAV strains circulating in 1933 or later (**Supplementary Fig. 4**). Given the size of this fragment and that of the fragments initially targeted to characterize the BM PA sequence[2], this apparent recombination may reflect the contamination of one of the PCR reactions that allowed for the reconstruction of the BM genome with a PCR product derived from a later influenza A virus strain. However, we cannot formally exclude that the BM PA fragment is a genuine but rare case of natural recombination[3] or that it represents a sequencing artefact due to intrahost variation. Resequencing of the BM genome using PCR-independent methods should clarify this issue.

In light of the hypothesis of an avian origin of the pandemic virus, we investigated the presence of amino acid (aa) signatures known or suspected to be associated with avian-to-human adaptation, either because they have been characterized functionally or found to be distributed differentially across bird- and human-infecting influenza viruses. Here, we focused on the high quality (but imprecisely dated) genome derived from MU-162 and identified two such positions. In PB2, we found a M631L aa change within the PB2 627 host range domain. Although this mutation has recently been described as a main mediator of adaptation and lethality of an avian influenza virus in mice [4], all but one human H1N1 strains (including all other 1918 influenza viruses) present a methionine at this position, suggesting that this change did not have profound evolutionary implications. On the contrary, we found that MU-162 has a leucine (L) at position 61 in NP, which is the residue most commonly found in human H1N1 strains prior to the 2009pdm (**Supplementary Fig. 5**), whereas an overwhelming majority of avian influenza A viruses present an isoleucine (I) at this position [5]. Interestingly, MU-162 was the only 1918 influenza virus presenting the I61L change, indicating this mutation only reached fixation after the pandemic peak.

Supplementary Note 4: Phylogenetic analyses on 1918 viruses

Evidence for multiple transatlantic migrations

Analyses of the HA with and without ambiguity at alignment positions 356 and 1600 show that the A/London/1/1918 sequence, sampled on 1918-11-13 and representing the first wave of the epidemic in the 1918-1919 winter in London, is most likely not from the same pandemic lineage that caused the second wave of the epidemic that winter (represented by A/London/1/1919, sampling date: 1919-02-15) (.75 and .73 PP). This view is reinforced by a separate analysis of the 1918 HA clade. For this we assumed an exponential growth model and a strict clock and specified an informative normal prior distribution on the evolutionary rate parameter. Specifically, the mean of this prior distribution was set to the mean of the estimate of the evolutionary rate in human hosts estimated in the HSLCext model, and the standard deviation was set so that the 2.5 and 97.5 percentiles of the normal prior corresponded to those of the estimate of the evolutionary rate in human hosts estimated in the HSLCext model. In these analyses, posterior support for different epidemic lineages in the first and second wave in London in the 1918-1919 winter increases to .88. Like the London samples, the isolates from Germany cluster in between pandemic variants sampled in North America (**Fig. 3** and **Supplementary Fig. 6**). This is in line with extensive transatlantic mixing of pandemic influenza. To more explicitly assess support for a migration scenario, the relative fit of models in which the clustering of the European isolates was not constrained versus constrained to be monophyletic was determined using the Generalised Stepping Stone sampling. In line with the clustering patterns, the unconstrained model clearly better fits the pandemic clade, irrespective of the use of ambiguity characters in the BE-576 sequence (ln BF 10.7 and 8.9). This agrees with the rejection of the constrained topology according to the AU-test[6] as implemented in IQtree[7] (p-value 0.0097 and 0.0087). The AU-test also rejects the topology in which monophyly is enforced for the pandemic peak viruses (p-value 0.0132 and 0.0091).

Similar epidemic dynamics in 1918 and 2009

To test whether the 1918 pandemic dynamics are shared with those of later influenza pandemics, we attempted to compile a data set with spatiotemporal spacing matching that of the 1918 pandemic as closely as possible for the 1957 H2N2, 1968 H3N2 and 2009 H1N1 pandemics. Unfortunately, only for 2009H1N1pdm there is a sufficient number of well-

annotated sequences from samples obtained during its emergence such that a sample resembling that of the 1918 pandemic can be selected. For this, the number of days between the earliest available and subsequent 1918 samples was approximated as closely as possible, starting from the earliest 2009H1N1pdm isolate (EPI-ISL-103102 obtained on 2009-03-03). 2009H1N1pdm isolates with temporal spacing matching the corresponding 1918 isolate were kept when the country or continent of sampling also agreed. When this was not the case, a 2009H1N1pdm isolate from the same country or continent as the corresponding 1918 sample obtained as closely as possible in time was selected. Of the 21 1918 HA-samples, two have sampling date uncertainty. For the sampling time difference calculation for A/Brevig_Mission/1/1918 (sampled in November 1918), its date was set to November 1st. For MU-162, of which we only know the sampling year, the sampling date for the calculations was randomly drawn from a uniform distribution spanning the appropriate uncertainty. An overview of the selected 2009H1N1pdm sample and its correspondence with the 1918 HA-sample is available upon request.

The same evolutionary models as for the 1918 HA-clade analyses were specified for the 2009H1N1pdm dataset. As for the 1918 pandemic we find strong support against monophyletic clustering of European lineages (ln BF 16.2). Likewise, the 2009H1N1pdm data indicate that several early lineages kept co-circulating over the time frame of the available sample of 1918 pandemic viruses. Both types of constraints were also rejected by the AU-test[68] as implemented in IQtree[7] (p-value for monophyly of European isolates 0.00299; p-value for monophyly of the isolates sampled in the time frame that corresponds to the 1918 pandemic peak viruses: 0.00773).

Reconciling clock and non-clock topologies

Non-clock maximum likelihood phylogenetic reconstruction indicates that human seasonal H1N1 and 1918 pandemic viruses cluster together with reasonably high bootstrap support, with the seasonal lineage nested within the 1918 pandemic variants, for both the HA and NA segments (**Supplementary Fig. 7**), while inference under the standard HSLC model places the human seasonal lineage as a sister clade of the classical swine flu and 1918 pandemic lineages (**Supplementary Fig. 8a**).

If the human seasonal and pandemic lineages are indeed monophyletic, the incompatible pattern under the standard HSLC model - which assumes a constant rate of evolution in each of the host-specific lineages - could be induced by a considerably higher rate of evolution in the years following the pandemic. This would result in a considerably higher divergence between

pandemic and seasonal viruses for H1 and N1 than expected under a strict clock, and could therefore induce a sister lineage pattern with a relatively deep MRCA in the time-measured reconstructions. To explore this hypothesis, phylogenies including only the human H1 and N1 taxa were estimated using the same substitution and demographic models as under the standard HSLC but specifying a relaxed clock model [8].

For HA, this indicates an elevated evolutionary rate on the branch ancestral to the human seasonal MRCA, and results in a topology that is compatible with the non-clock ML phylogeny (**Supplementary Fig. 9**). For NA, a pattern similar to that of HA emerges (**Supplementary Fig. 9**). These results are in line with the idea that significant divergence that accumulated after 1918-1919 could indeed induce the sister pattern under a constant rate assumption over the entire lineage.

Simulation-based assessment of standard and extended host-specific local clock model inference and non-clock phylogenetic inference.

The simulations under the extended host-specific local clock (HSLCext) pattern (**Supplementary Fig. 10a**), as estimated for the HA segment, indicate that the non-clock ML tree reconstructions correctly infer a monophyletic clade for the 1918 pandemic and human seasonal virus in 17 out 20 cases, and incorrectly infer a deep TMRCA in 3 out of 20 replicates (bottom two panels). Not accommodating the higher rate on the branch ancestral to human seasonal results in a biased inference of a deep TMRCA in 17 out of 20 replicates for the standard HSLC (HSLCstd) model.

The simulations under the HSLCstd pattern (**Supplementary Fig. 10b**), as estimated for the HA segment, indicate that both the non-clock ML tree reconstruction as well as the HSLCext model, which allows for a different rate on the branch ancestral to human seasonal, are able to recover the right topology with a 0.95 probability. In line with this, the extended HSLC model does not infer a significantly positive effect on the branch ancestral to human seasonal (not shown). This indicates that the extended HSLC model and the non-clock ML inference are unlikely to produce biased results.

HSLC standard versus HSLC extended: impact on clustering of human seasonal H1N1

For each segment, the plausibility that the seasonal lineage directly emerged from the pandemic diversity increases under the extended HSLC model as compared to under the standard HSLC

model (**Supplementary Fig. 8b**). This is particularly outspoken for the HA segment: where the results under the standard HSLC model are in line with reassortment between the human pandemic lineage and an antigenically distinct co-circulating H1 virus [9], the clustering patterns under the extended HSCL model suggest otherwise. Support for different evolutionary trajectories of the human seasonal lineage under the HSLCext model varies depending on the inclusion of short HA sequence fragments of pandemic isolates, pointing to a need for more (near) full length HA sequences from around the time of the pandemic. For NP and PA, we find perfect support for nested clustering of the seasonal taxa within the diversity of their pandemic counterparts under both clock models. Support for this is high to perfect for MP, NS and PB2, and is low to moderate for NA and PB1.

To further investigate the fit of the two models, we formally compared the fit of the HSLCstd and HSLCext model to the HA data using generalised stepping-stone sampling (Baele et al., Sys Biol 2016). In line with the clear support for an additional rate effect on the branch that is ancestral to the human seasonal diversity, we find good support for the HSLCext over the HSLCstd model (**Supplementary Table 3**).

	w/ ambig	w/o ambig
a. HSLCstd	-35798.49	-35812.87
b. HSLCext	-35790.82	-35801.82
Bayes factor (b - a)	7.67	11.05

Supplementary Table 3. Support for the HSLCext model. The log marginal likelihood estimates for both models are given in rows a and b. Bayes factors are calculated as the ratio of the marginal likelihoods of both models. Because the likelihoods are expressed as log-likelihoods, this equates to taking the difference of the log-likelihood of competing models.

Finally, we also ran the HSLCext model on the segment data sets without the new 1918 sequences from Germany. As with the complete data set, the inclusion probability for a rate effect on the human seasonal stem branch is 1 for all segments. In addition, the rate effect on the human seasonal stem branch is of the same magnitude as with the complete data set (**Supplementary Fig.11**). Although the addition of the new German sequences did not impact the outcome of this specific analysis, generating these genomes led to revisiting, and ultimately improving, the HSLCstd model.

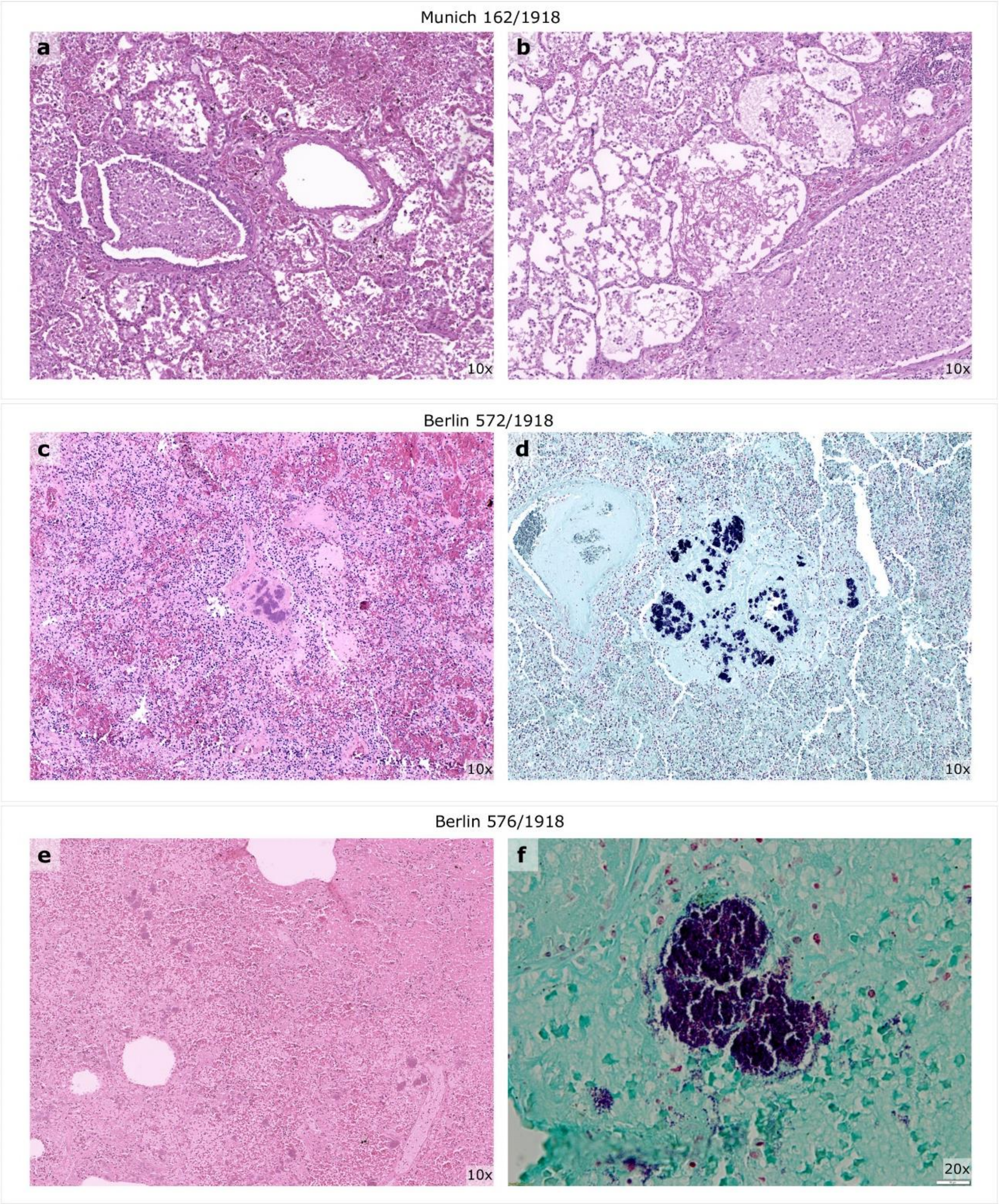

Supplementary Fig. 1. Histopathological findings of influenza-positive lung specimens. (**a**) and (**b**) display H&E staining of sample MU-162, (**c**) and (**d**) show H&E and gram staining for sample BE-572, respectively, (**e**) and (**f**) show H&E and gram staining for sample BE-576, respectively. Images were taken using either a 10X or 20X objective, which corresponds to a 100X or 200X magnification, respectively. For each sample, at least three different areas were examined histopathologically.

a

b

c

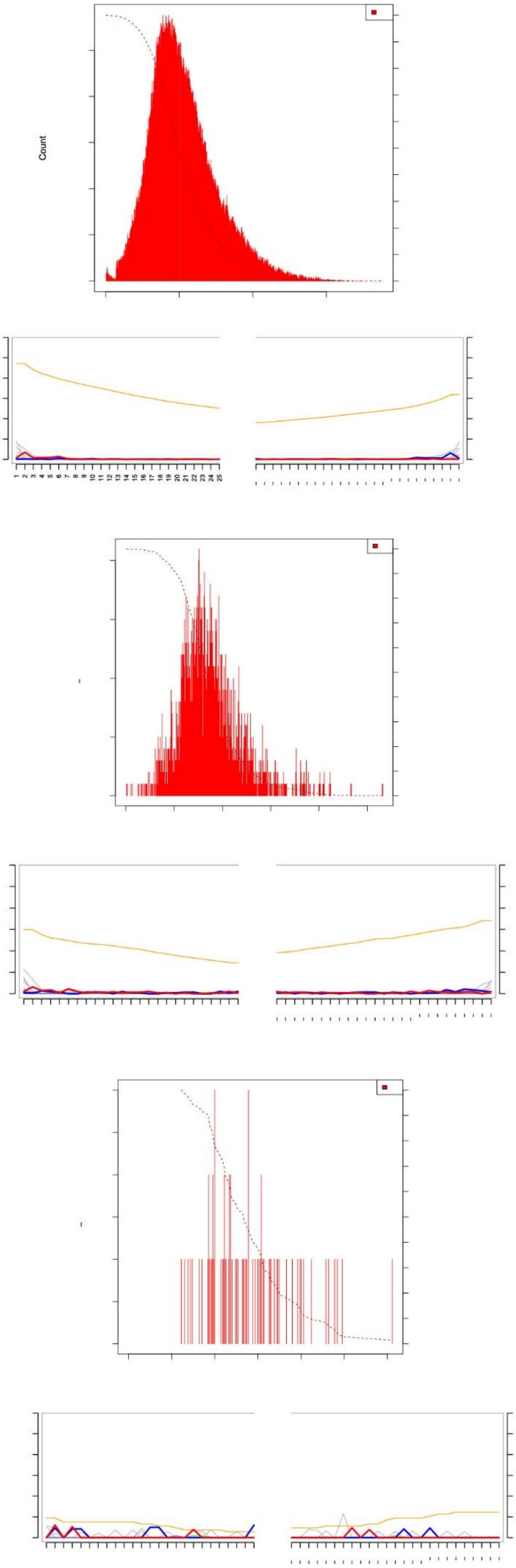

Count
Frequency
0.30
0.25
0.20
0.15
0.10
0.05
0.00
1 2 3 4 5 6 7 8 9 10 11 12 13 14 15 16 17 18 19 20 21 22 23 24 25

Supplementary Fig. 2. Insert size distribution (upper panel) and mapDamage profile (lower panel) of reads in individual libraries containing influenza sequences. (**a**) Library number 162_9 (MU-162). (**b**) Library number 572_1 (BE-572). (**c**) Library number 576_1 (BE-576).

Library	Mapping reference	Median insert size (nt)	Maximum insert size (nt)
**162_1**	MU-162/1918	119.5	403
**162_2**	MU-162/1918	118.5	200
**162_3**	MU-162/1918	132.5	320
**162_4**	MU-162/1918	133.5	236
**162_5**	MU-162/1918	133	318
**162_6**	MU-162/1918	112.5	184
**162_7**	MU-162/1918	114.5	313
**162_8**	MU-162/1918	115	337
**162_9**	MU-162/1918	199	751
**572_1**	MU-162/1918	166	532
**572_1**	MU-162/1918	173	453
**572_3**	MU-162/1918	201.5	381
**572_4**	MU-162/1918	165	166
**576_1**	MU-162/1918	133.5	306
**576_2**	MU-162/1918	151	194
**576_3**	MU-162/1918	133	281
**576_5**	MU-162/1918	102	102
**576_6**	MU-162/1918	113	113
**576_9**	MU-162/1918	173.5	322

Supplementary Fig. 3. Median and maximum insert sizes for reads mapping to the 1918 influenza MU-162 genome. Shown are the values for the individual libraries generated for the three samples containing influenza reads.

Gene	Strain	nt change	aa position	aa change
**HA**	BE-576	G356A	119	R119K
	BE-576	T1600C	534	Y534H
	CU	G715A	239	D239N
	CU	G472A	158	A158T
**M1**	BM	C10T	4	N
	BE-572, MU	G543A	181	N
	BE-572, MU	C693T	231	N
	BM	C700A	234	L234I
**M2**	BM	T849G	54	L54R
**NA**	MU	A435G	145	N
	BM	T462C	154	N
	BE-572, MU	A588C	196	N
	BM	C768A	256	F256L
	MU	A774C	258	N
	BM	C900T	300	N
	MU	A1036G	346	I346V
	MU	G1386A	462	N
	BM	C1397G	466	T466S
**NP**	BE-572, BE-576	A47G	16	D16G
	BE-572, BE-576	G165A	55	N
	MU	A181T	61	I61L
	BM	G504A	168	N
	BE-572, BE-576 (only 2x coverage)	C848T	283	P283L
	MU	A864G	288	N
	BM	G987A	329	N
	MU	C1221T	407	N
	BM	T1488C	496	N
**NS1**	MU	T64G	22	F22V
	BM	G224A	75	G75E
**PA**	BE-572, BE-576	T194C	65	S65F
	BM	A453T*	151	N
	BM	A456G*	152	N
	BM	G473A*	158	R158K°
	BM	A495G*	165	N
	BM	A498G*	166	N
	BM	T501C*	167	N
	BM, CU	A510G*	170	N
	BM	G522A*	174	N
	MU	C531T	177	N
	MU, BE-572	G1009T	337	A337S
	MU	G1203A	401	N
	CU	C1548T	516	N
	BE-572	T1557C	519	N
	MU	C1709T	570	T570I
	BM	C1710A	570	N
**PB1**	BM	A161G	54	K54R
	MU, BE-572, BE-576	A1164G	388	N
	MU	A2081G	694	N694S
**PB1-F2**	MU	G2202A	23	D23N
**PB2**	MU	A12G	4	I4M
	MU, BE-572, BE-576	C246T	82	N
	MU, BE-572, BE-577	G322A	108	A108T
	MU, BE-572	A552G	184	N
	MU, BE-572	A582G	194	N
	MU, BE-572	A639C	213	N
	MU, BE-572	G816A	272	N
	BE-572	T1013A	338	V338D
	MU, BE-572	A1038G	346	N
	BE-572	A1140G	380	N
	MU	G1326A	442	N
	MU	A1452T	484	N
	MU	G1615A	539	V539I
	MU	A1891T	631	M631L

Supplementary Fig. 4. Nucleotide and amino acid changes in the pandemic strains analysed. Nucleotide (nt) and amino acid (aa) changes are reported with reference to the position in the coding sequence of the MU-162 strain (here referred to as MU). Each nt/aa change is reported as nt/aa observed in the other strains followed by the position and then the nt/aa observed in the strain that is different. N stands for no aa change conferred. * indicate nt differences identified in the potential recombinant fragment of the BM sequence. °Aa change R158K is also present in PA-X. No nt change was present in NS2.

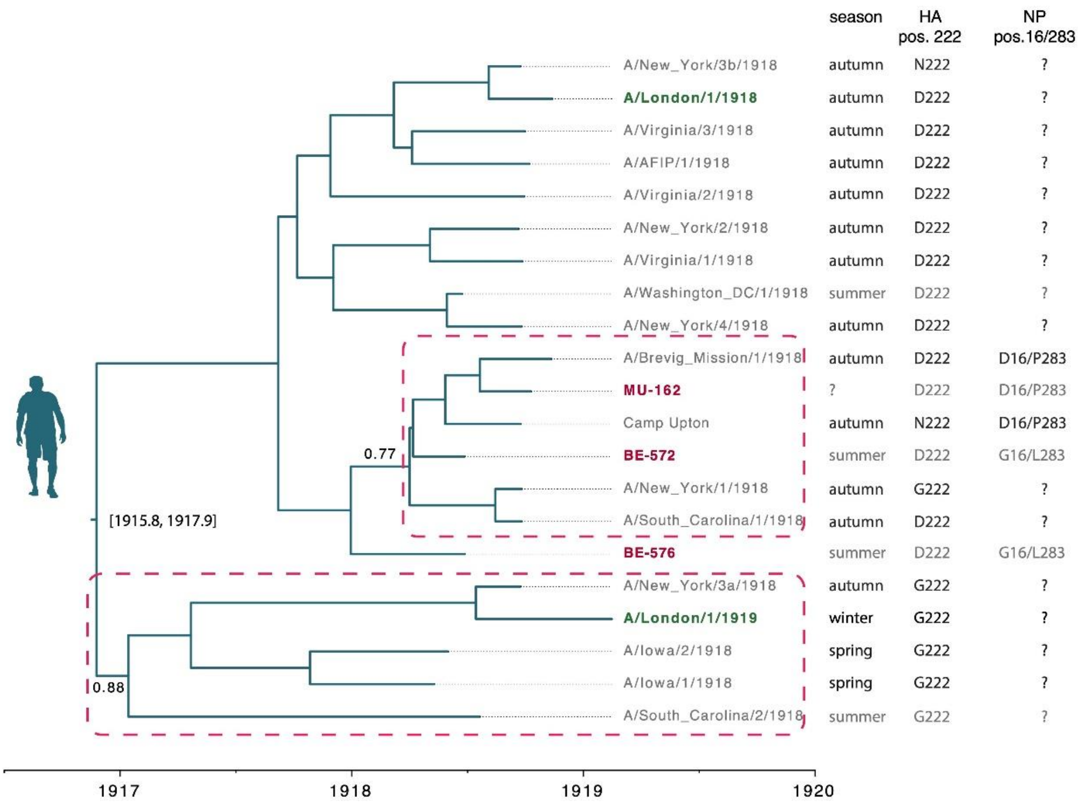

Supplementary Fig. 5. Time-scaled phylogeny reconstructed based on HA sequences from 1918 flu strains. US strains are in light grey, European strains are in dark red (Germany) and dark green (UK). Dashed rectangles highlight clades comprising strains from different continents and with posterior support ≥ .75; for these clades posterior probabilities are reported above stem branches. The season column indicates from which season the samples originate, with light grey for the pre-pandemic peak period (spring and summer) and dark grey for the pandemic peak period (autumn and winter). The amino acid residues at HA position 222 and NP positions 16 and 283 are also indicated. '?' indicate the absence of information. Numbers between brackets next to the root node indicate the 95%HPD of its estimated age. For the BE-576 sequence, a majority rule consensus base calling was used in this analysis.

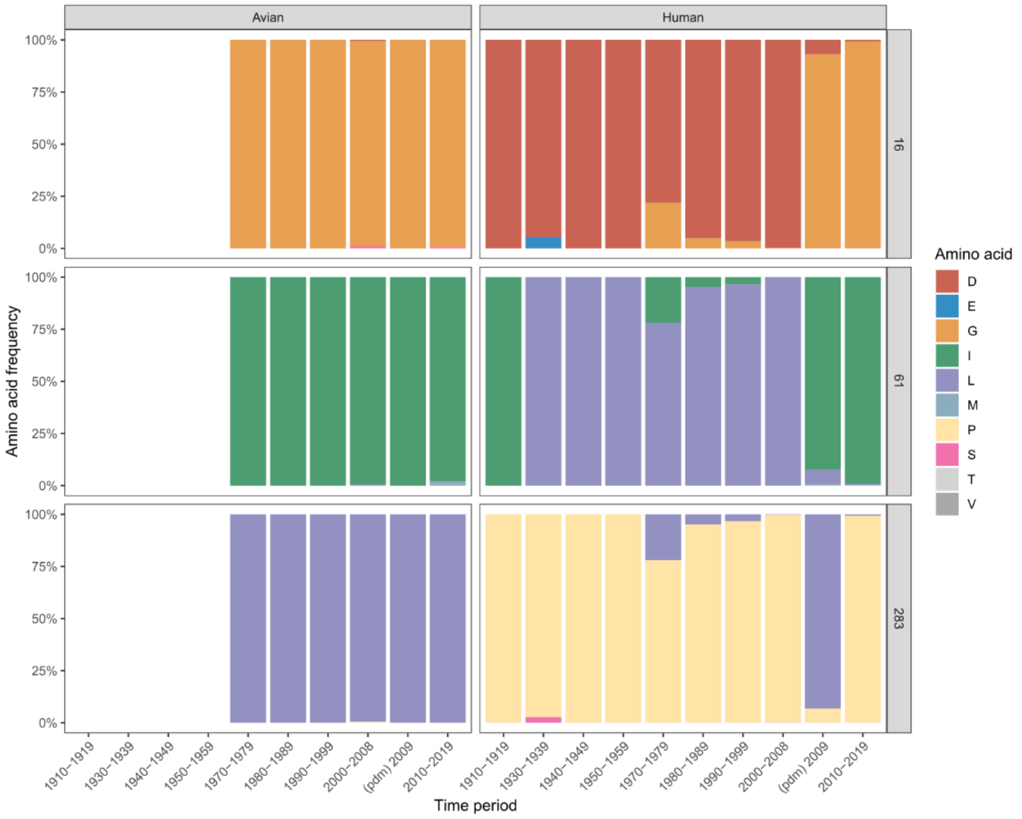

Supplementary Fig. 6. Amino acid frequency in the NP protein of human and avian H1N1 strains sorted by decade. Displayed are the amino acid positions discussed in the main text (16, and 283) and in the Supplementary Information (61). Data source: Influenza Virus Database @NCBI, Filter: Complete NP sequences, H1N1, human versus avian. Downloaded on 2020-05-04**.** 1918 IAV sequences were not included.

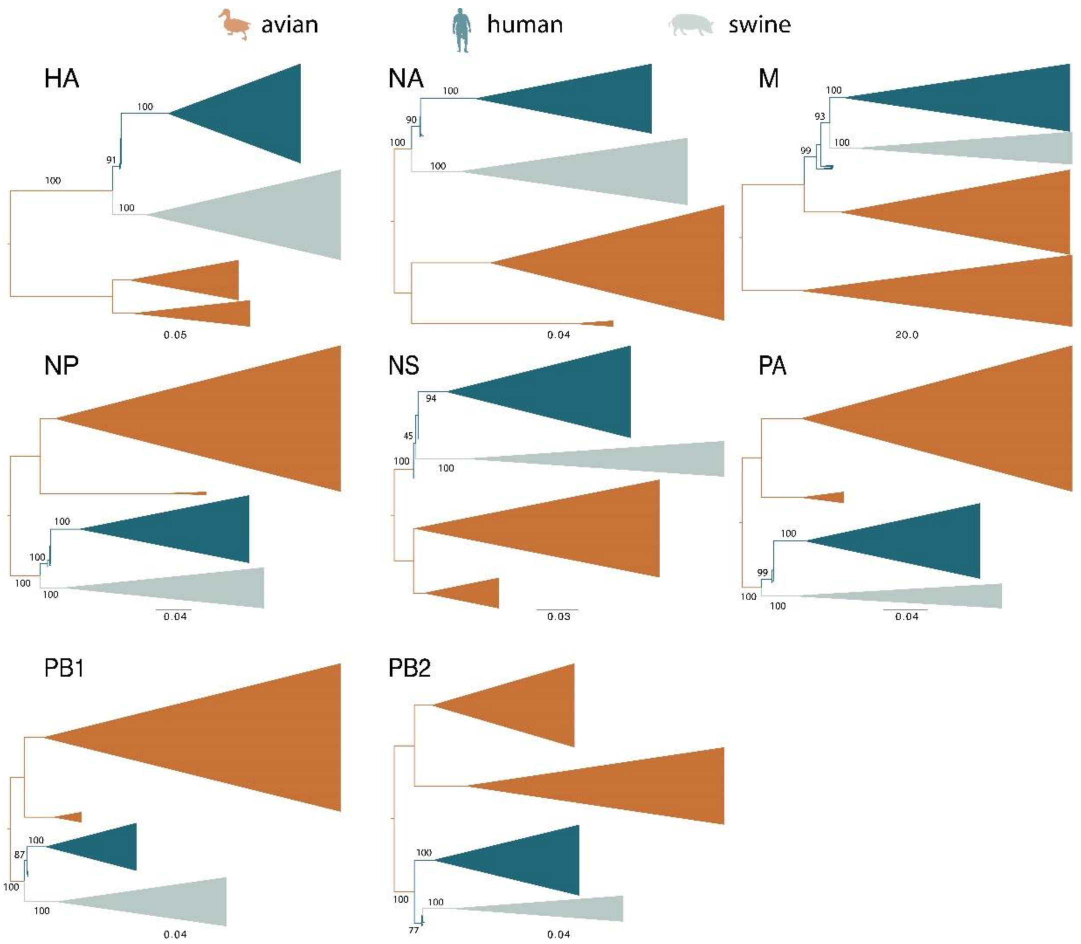

Supplementary Fig. 7. Maximum likelihood phylogenetic reconstructions for all segments. Lineages are colored according to host and numbers at the major nodes of interest represent the percentage bootstrap support.

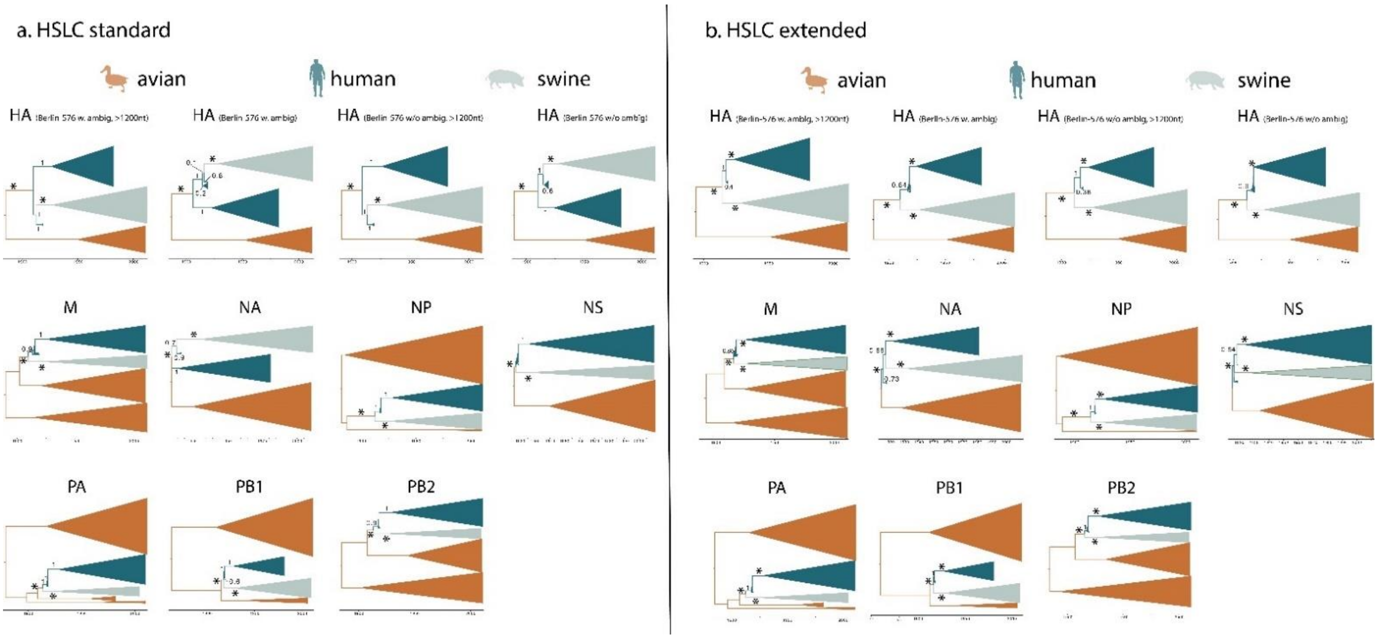

Supplementary Fig. 8. Illustration of the rate category specification. The trees represent maximum clade credibility (MCC) summary trees for each segment. The correspondence between branch colors and host category is as in the legend. Asterisks indicate nodes for which a monophyly constraint was specified in order to maintain identifiability of the model. Numbers next to branches refer to their posterior support. **a,** MCC trees inferred under the standard HSLC model. **b**, MCC trees inferred under the extended HSLC model. For HA, '>1200nt' indicates that only human pandemic taxa of length > 1200 nt were considered.

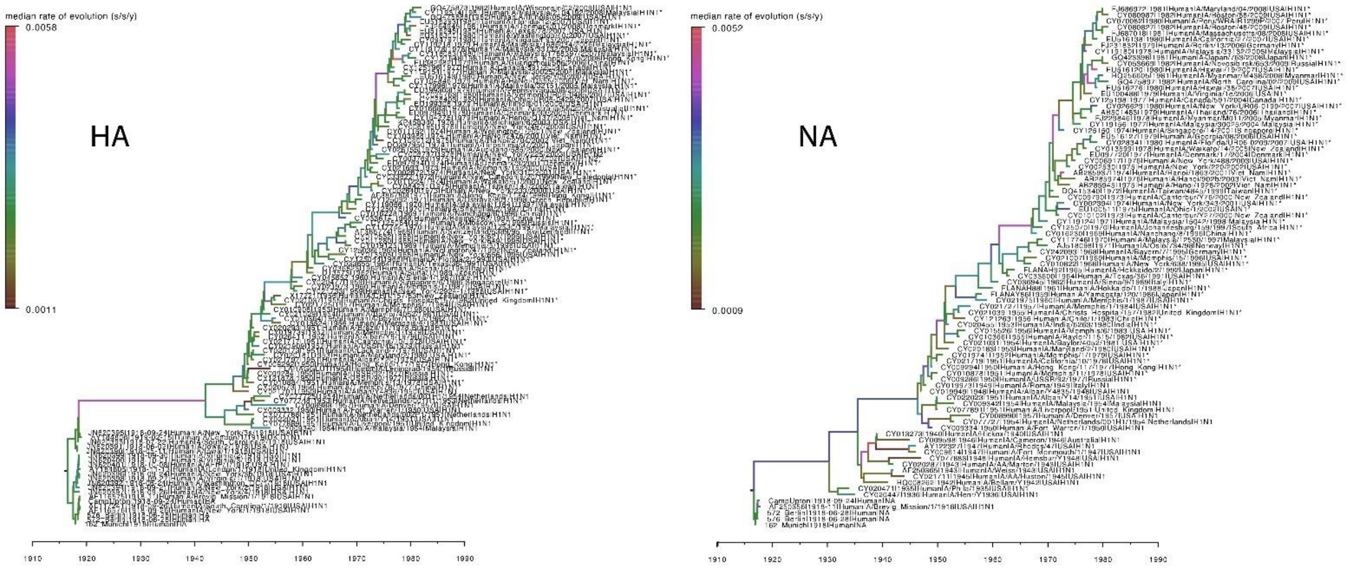

Supplementary Fig. 9. MCC summary trees of human H1 and N1 inferred under a relaxed clock model. Branches are colored according to the median of the rate of evolution estimated over that branch. Correspondence between branch colors and the rate of evolution is as in the legend. For HA, the BE-576 genome with ambiguities was used.

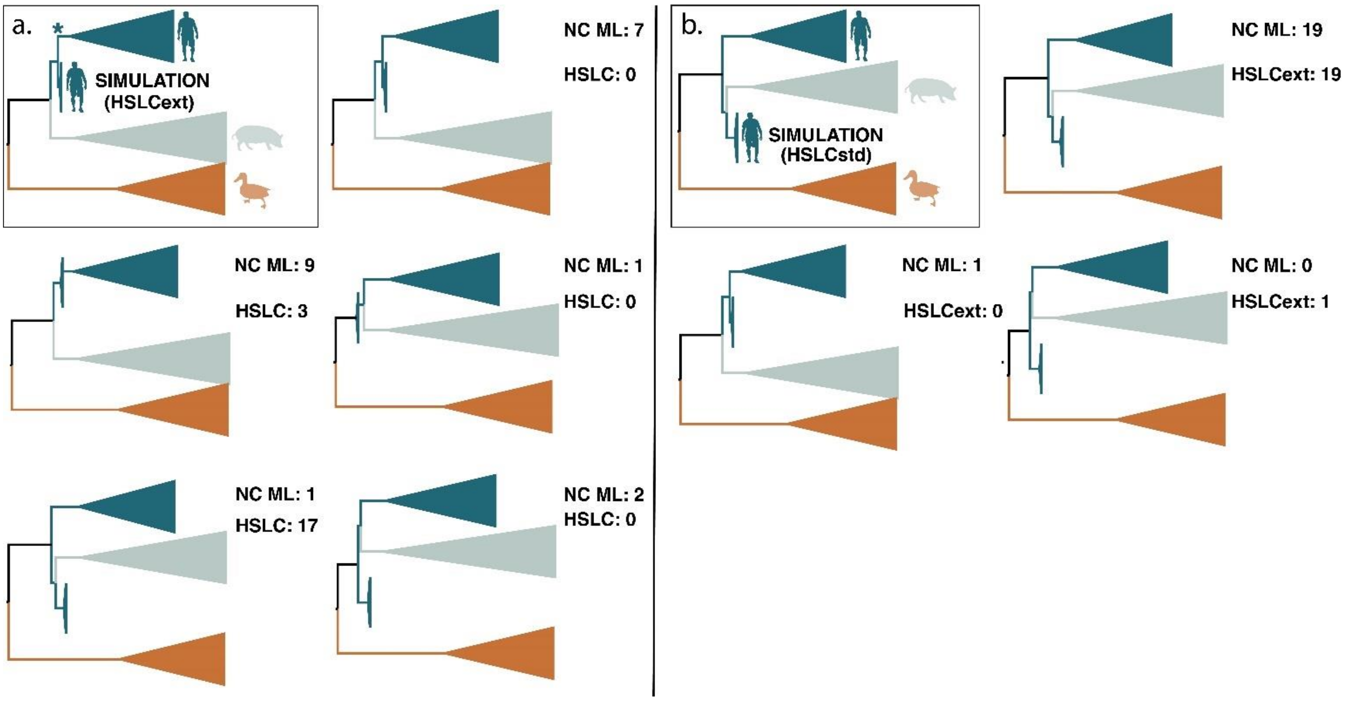

Supplementary Fig. 10. a, Simulations under the extended host-specific local clock (HSLCext) model estimate for the HA segment. The top left time-measured phylogenetic tree with a grey background represents the estimate under the HSLCext model for HA. A star denotes the branch that is allowed to have a separate rate relative to the standard HSLC model (HSLCstd). 20 data sets were simulated under this scenario and both non-clock maximum likelihood (NC ML) phylogenetic estimation and BEAST estimation using the HSLCstd model were performed on the replicate data. Five topologies are used to summarize the estimates for both methods. **b, Simulations under the standard host-specific local clock (HSLCstd) model estimate for the HA segment.** The top left time-measured phylogenetic tree in a black box represents the estimate under the standard HSLC model for HA. 20 data sets were simulated under this scenario and both non-clock maximum likelihood (NC ML) phylogenetic estimation and BEAST estimation using the extended HSLC (HSLCext) model were performed on the replicate data. Three topologies are used to summarize the estimates for both methods.

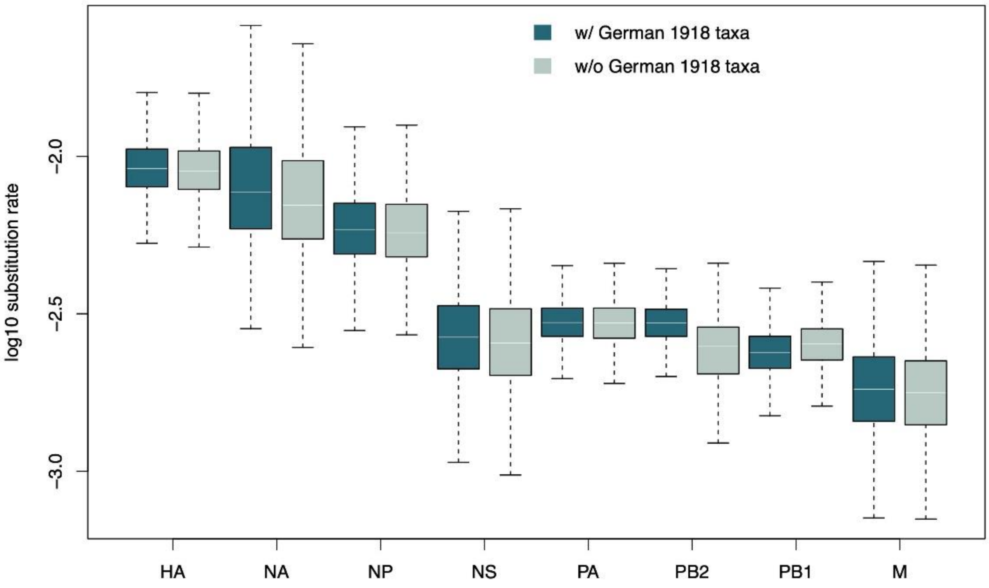

Supplementary Fig. 11. Evolutionary rate estimates for the human seasonal ancestral branch for each segment. Segments are ordered as in Figure 4 panel C of the main manuscript. The dark and light green colored boxplots represent segments' estimates including the new 1918 German sequences (dark) or not (light). The horizontal line in the whisker plots represents the mean. The lower and upper bounds of the boxes indicate the first and third quartile, respectively. Vertical lines are the upper and lower whisker representing the minimum of the largest value and 1.5 times the inter quartile distance, respectively the maximum of the smallest value and 1.5 times the inter quartile distance. Sample size from the MCMC chain including the German taxa is 2702 for NA, and 4502 for all other segments. Sample size from the MCMC chain excluding the German taxa is 2950 for NA, and 4500 for all other segments Source data are provided as a Source Data file.

References

1. Cliver, D. O. Capsid and Infectivity in Virus Detection. *Food Environ. Virol.* **1**, 123–128 (2009).
2. Taubenberger, J. K. *et al.* Characterization of the 1918 influenza virus polymerase genes. *Nature* **437**, 889–893 (2005).
3. Han, G. Z. & Worobey, M. Homologous recombination in negative sense RNA viruses. *Viruses* **3**, 1358–1373 (2011).
4. Zhang, X. *et al.* Enhanced pathogenicity and neurotropism of mouse-adapted H10N7 influenza virus are mediated by novel PB2 and NA mutations. *J. Gen. Virol.* **98**, 1185–1195 (2017).
5. Chen, G. W. *et al.* Genomic signatures of human versus avian influenza A viruses. *Emerg. Infect. Dis.* **12**, 1353–1360 (2006).
6. Shimodaira, H. An approximately unbiased test of phylogenetic tree selection. *Syst. Biol.* **51**, 492–508 (2002).
7. Nguyen, L. T., Schmidt, H. A., Von Haeseler, A. & Minh, B. Q. IQ-TREE: A fast and effective stochastic algorithm for estimating maximum-likelihood phylogenies. *Mol. Biol. Evol.* **32**, 268–274 (2015).
8. Drummond, A. J., Ho, S. Y. W., Phillips, M. J. & Rambaut, A. Relaxed phylogenetics and dating with confidence. *PLoS Biol.* **4**, 699–710 (2006).
9. Worobey, M., Han, G. Z. & Rambaut, A. Genesis and pathogenesis of the 1918 pandemic H1N1 influenza a virus. *Proc. Natl. Acad. Sci. U. S. A.* **111**, 8107–8112 (2014).
