# Supplementary material for: Archival influenza virus genomes from Europe reveal genomic variability during the 1918 pandemic
Source: Nat Commun. 2022 May 10;13:2314. doi: 10.1038/s41467-022-29614-9 (PMC9090925; doi:10.1038/s41467-022-29614-9)
Supplement: Supplementary file 3 — Reporting Summary [file 41467_2022_29614_MOESM3_ESM.pdf]

nature research

Corresponding author(s): Sebastien Calvignac-Spencer

Last updated by author(s): Jan 10, 2022

Reporting Summary

Nature Research wishes to improve the reproducibility of the work that we publish. This form provides structure for consistency and transparency in reporting. For further information on Nature Research policies, see Authors & Referees and the Editorial Policy Checklist .

Statistics

For all statistical analyses, confirm that the following items are present in the figure legend, table legend, main text, or Methods section.

n/a	Confirmed	
☐	☒	The exact sample size (n) for each experimental group/condition, given as a discrete number and unit of measurement
☐	☒	A statement on whether measurements were taken from distinct samples or whether the same sample was measured repeatedly
☐	☒	The statistical test(s) used AND whether they are one- or two-sided<br>*Only common tests should be described solely by name; describe more complex techniques in the Methods section.*
☐	☒	A description of all covariates tested
☒	☐	A description of any assumptions or corrections, such as tests of normality and adjustment for multiple comparisons
☐	☒	A full description of the statistical parameters including central tendency (e.g. means) or other basic estimates (e.g. regression coefficient) AND variation (e.g. standard deviation) or associated estimates of uncertainty (e.g. confidence intervals)
☐	☒	For null hypothesis testing, the test statistic (e.g. F, t, r) with confidence intervals, effect sizes, degrees of freedom and P value noted<br>*Give P values as exact values whenever suitable.*
☐	☒	For Bayesian analysis, information on the choice of priors and Markov chain Monte Carlo settings
☒	☐	For hierarchical and complex designs, identification of the appropriate level for tests and full reporting of outcomes
☒	☐	Estimates of effect sizes (e.g. Cohen's d, Pearson's r), indicating how they were calculated

Our web collection on statistics for biologists contains articles on many of the points above.

Software and code

Policy information about availability of computer code

Data collection	No software was used
Data analysis	Raw reads were pre-processed using Trimmomatic v0.36 with the following settings: LEADING:30 TRAILING:30 SLIDINGWINDOW:4:40 MINLEN:40. Reads were initially classified using Kraken2. Trimmed reads were then mapped using BWA-MEM v0.7.15-r1140. De novo assembly using metaSPAdes v3.12.0. Upon trimming, paired end reads were merged using the Clip&Merge tools from EAGER. We sorted mapping files and removed duplicates using the SortSam and MarkDuplicates tools from Picard (http://broadinstitute.github.io/picard). We assessed RNA damage using mapDamage v2. The software Geneious v10.0.5 was used for visualization of NGS data. Recombination analyses for the PA segment were run in Rdp4 v4.97.We reduced the dataset to unique sequences using FaBox v1.6. We ran Rdp4 with all methods (changing the settings of the bootscan approach to a window size of 100). PhyML37 was finally run on the backbone and recombinant region. Sequences were aligned with MAFFT v7.313 and Aliview v1.19 was used for manual refinement of the alignments. Maximum likelihood (ML) trees for all datasets were reconstructed using IQTREE v2.0. Bayesian analyses were run in BEAST v1.10. We used BEAGLE 3 for efficient likelihood computation and simulate the MCMC chains sufficiently long to ensure stationarity and mixing as diagnosed using Tracer v1.7.0. We summarized posterior tree distributions in the form of maximum clade credibility (MCC) trees and visualize these trees using FigTree v1.4.4 (http://tree.bio.ed.ac.uk/software/figtree/). Selection analyses were performed using codon substitution model approaches implemented in HyPhy v2.5.17. Specifically, we tested for episodic (diversifying) selection and relaxed selection along the branch ancestral to human seasonal viruses in the human H1N1 lineage using aBSREL and RELAX v2.5.17, respectively (both available in HyPhy). Simulations were performed using piBUSS v1.4. In agreement with the ethics committee of the Charité, Berlin, human reads were removed from the sequencing files prior to uploading them to the European Nucleotide Archive. Filtered reads were mapped to the human RefSeq genome assembly GRCh38.p13 (GCF_000001405.39) using BWA-MEM v0.7.15-r1140. Unmapped reads were extracted using SAMtools v1.3.1 and then extracted by name from the original fastq files using seqtk (https://github.com/lh3/seqtk).

For manuscripts utilizing custom algorithms or software that are central to the research but not yet described in published literature, software must be made available to editors/reviewers. We strongly encourage code deposition in a community repository (e.g. GitHub). See the Nature Research guidelines for submitting code & software for further information.

Data

Policy information about availability of data

All manuscripts must include a data availability statement. This statement should provide the following information, where applicable:

- Accession codes, unique identifiers, or web links for publicly available datasets
- A list of figures that have associated raw data
- A description of any restrictions on data availability

Other relevant data supporting the findings of the study are available in this published article and its Supplementary Information files. All raw reads generated for this study have been deposited to the European Nucleotide Archive under project number PRJEB41631 (sample numbers ERS5447402-413, ERS6621911, and ERS5549335-ERS5549361). Human reads were removed prior to uploading. Kraken2 results for all specimens can be visualized through Krona plots at https://zenodo.org/record/4384755. Nucleotide alignments for the 8 viral segments are available at https://zenodo.org/record/4384715.

Field-specific reporting

Please select the one below that is the best fit for your research. If you are not sure, read the appropriate sections before making your selection.

☒ Life sciences ☐ Behavioural & social sciences ☐ Ecological, evolutionary & environmental sciences

For a reference copy of the document with all sections, see nature.com/documents/nr-reporting-summary-flat.pdf

Life sciences study design

All studies must disclose on these points even when the disclosure is negative.

Sample size	In this study we included all available archival samples recovered from museum collections (n=13).
Data exclusions	No data was excluded
Replication	Each sample was processed and sequenced independently at least 4 times to confirm influenza virus presence. The amount of reads mapping to influenza virus varied across experiments but overall viral presence was confirmed in all independent experiments.
Randomization	Randomizaion is not applicable to this study, which is aimed at investigating infectious causes of disease in archival samples selected based on pathology reports.
Blinding	Blindingis not applicable to this study, which is aimed at investigating infectious causes of disease in archival samples selected based on pathology reports.

Reporting for specific materials, systems and methods

We require information from authors about some types of materials, experimental systems and methods used in many studies. Here, indicate whether each material, system or method listed is relevant to your study. If you are not sure if a list item applies to your research, read the appropriate section before selecting a response.

Materials & experimental systems

n/a	Involved in the study	
☒	☐	Antibodies
☒	☐	Eukaryotic cell lines
☒	☐	Palaeontology
☒	☐	Animals and other organisms
☐	☒	Human research participants
☒	☐	Clinical data

Methods

n/a	Involved in the study	
☒	☐	ChIP-seq
☒	☐	Flow cytometry
☒	☐	MRI-based neuroimaging

Human research participants

Policy information about studies involving human research participants

Population characteristics	The samples used derive from humans deceased in Germany and Austria between 1900 and 1931.
Recruitment	Recruitment is not applicable because the study was based on archived human biological specimens for the early 20th century.
Ethics oversight	Ethics approval was obtained from the ethics committee of the Charité (Berlin, Germany) under the reference number EA4/212/19.

Note that full information on the approval of the study protocol must also be provided in the manuscript.
